# Ketogenic Diet-Based Therapy for Fatigue in Patients with Multiple Sclerosis

**DOI:** 10.3390/nu18101496

**Published:** 2026-05-08

**Authors:** Francesca Filippi, Simone Lorenzut, Riccardo Garbo, Eleonora Lamon, Ilaria Del Negro, Annacarmen Nilo, Sara Pez, Gian Luigi Gigli, Mariarosaria Valente

**Affiliations:** 1 Department of Medicine (DMED), University of Udine, Via Colugna 50, 33100 Udine, Italy; annacarmen.nilo@gmail.com (A.N.); gianluigi.gigli@uniud.it (G.L.G.); mariarosaria.valente@uniud.it (M.V.); 2 Clinical Neurology Unit, Udine University Hospital, Piazzale Santa Maria della Misericordia, 33100 Udine, Italy; lamon.eleonora@spes.uniud.it (E.L.); delnegro.ilaria@gmail.com (I.D.N.); sara.pez91@gmail.com (S.P.); 3 Neurology Unit, Head Neck and Neuroscience Department, Santa Maria della Misericordia University Hospital, 33100 Udine, Italy; simone.lorenzut@asufc.sanita.fvg.it; 4 SC Neurology of Gorizia Monfalcone, Azienda Sanitaria Universitaria Giuliano Isontina (ASUGI), 34100 Gorizia, Italy; riccardo.garbo@outlook.it

**Keywords:** ketogenic diet, ketosis, ketones, multiple sclerosis, fatigue, sleepiness, sleep

## Abstract

**Background/Objectives:** Fatigue is a frequent, disabling and difficult-to-treat symptom of multiple sclerosis (MS). Low-grade inflammation and energetic dysfunction have been proposed as mechanisms underlying the pathogenesis of this symptom. Owing to its anti-inflammatory and metabolic properties, there is a rationale for ketogenic diet (KD) application in this setting. The aim of this study was to evaluate the effects of KD on fatigue and other frequently associated symptoms in a carefully selected group of patients with MS. **Methods**: We conducted a single-arm open-label interventional study on a strictly selected group of 16 non-obese patients with multiple sclerosis who were prescribed KD for three months. Fatigue, sleep quality, daytime somnolence, mood, and quality of life were assessed at baseline (T0), 1 month (T1), and 3 months (T3) using validated scales. **Results:** With respect to baseline, at 3 months we observed a significant reduction in Fatigue Severity Scale (5.18 ± 1.02 vs. 4.16 ± 0.98; *p* = 0.042), Epworth Sleepiness Scale (8.46 ± 3.05 vs. 5.64 ± 2.46; *p* < 0.001), Pittsburgh Sleep Quality Index (5.64 ± 3.53 vs. 7.62 ± 2.59; *p* = 0.009), Depression Anxiety Stress Scales-21 depression (3.18 ± 2.93 vs. 6.15 ± 3.81; *p* = 0.036) and anxiety (5.15 ± 4.10 vs. 1.55 ± 1.92; *p* = 0.019) sub-scales, and an improvement in energy sub-scale of multiple sclerosis Quality of Life-54 (52.49 ± 12.83 vs. 37.43 ± 14.26; *p* = 0.042). **Conclusions:** These findings suggest that KD might be useful for the treatment of fatigue, and they raise interest in the use of KD in the treatment of other symptoms frequently encountered in multiple sclerosis. Larger randomized controlled studies are needed to confirm these preliminary results.

## 1. Introduction

Multiple sclerosis is an inflammatory and demyelinating disease of the central nervous system, characterized by focal lesions mediated by the adaptive immune system and by progressive neurodegenerative processes [1]. MS affects approximately 2.8–2.9 million people worldwide [2] and more than 140,000 people in Italy, with approximately 3600 new diagnoses each year. The prevalence is around 227 cases per 100,000 inhabitants in mainland Italy and predominantly involves women, with a female-to-male ratio of about 3 to 1. Diagnosis typically occurs in young adulthood, between 20 and 40 years of age [3]. Fatigue is the most common chronic symptom in patients with MS, with an incidence of up to 90% of patients, often having a significant impact on everyday life activities [4,5]. Fatigue in MS is a multidimensional symptom distinct from normal tiredness and does not resolve with rest. It is a complex phenomenon, and it is complicated to define and identify. Typically, fatigue is classified as primary—directly related to the pathophysiological mechanisms of MS, including neuroinflammation, demyelination, and axonal energy failure—or secondary, arising from MS-related comorbidities such as depression, sleep disorders, pain, and deconditioning [6]. Regarding diagnostic criteria, the Multiple Sclerosis Council for Clinical Practice Guidelines (1998) defined clinically significant MS fatigue as fatigue present for any amount of time on ≥50% of days for more than 6 weeks, limiting functional activities or quality of life. For clinical and research purposes, the Fatigue Severity Scale (FSS)—with a mean score ≥ 4 across its nine items considered the threshold for pathological fatigue—is the most widely validated and used instrument [7,8]. The inclusion criterion of FSS ≥ 4 adopted in the present study is consistent with this established threshold.

The exact pathophysiology of MS-related fatigue is not fully understood, but it is thought to be multifactorial, involving both physical and psychological factors. Several mechanisms have been proposed: neuroinflammation with elevated pro-inflammatory cytokines (IL-6, TNF-α, IFN-γ) acting on central neural circuits; altered corticospinal and thalamocortical connectivity; mitochondrial dysfunction and impaired axonal energy metabolism, leading to so-called ‘virtual hypoxia’; and hypothalamic–pituitary–adrenal (HPA) axis dysregulation. These mechanisms are not mutually exclusive and likely act in concert [9,10].

Starting from the recognition of the so-called “sickness behavior” induced by pro-inflammatory cytokines, some observations suggest that inflammation may be one of the most relevant processes underlying fatigue [10]. Moreover, patients with conditions associated with fatigue, such as chronic fatigue syndrome and depression, may present altered levels of cytokines in the peripheral circulation, generally expressing a predominant Th1 pro-inflammatory profile [8]. Many studies have suggested higher levels of pro-inflammatory factors also in fatigued patients with MS (pwMS), although there are also some negative findings [4,11].

Another aspect of interest in fatigue pathogenesis is the possible existence of an energetic dysfunction in the CNS of MS fatigued patients, as suggested by some imaging [12,13] and laboratory findings. Demyelination in MS causes important changes from the neuronal energetic point of view, given that the loss of saltatory conduction determines a greater need for ATP consumption by Na+/K+ ATPase. In addition, the mitochondrial damage secondary to the inflammatory process contributes to creating this unfavorable energetic state defined as “virtual hypoxia” [9,14,15,16].

Disease-modifying therapies (DMTs) are currently the gold standard for the treatment of MS, and their effectiveness has been assessed through randomized clinical trials (RCTs). However, there is limited information regarding the impact of DMTs on fatigue in pwMS [17]. Consequently, increasing attention has been directed toward non-pharmacological interventions aimed at improving fatigue and quality of life in pwMS [18].

Considering the hypothesized physiopathology of MS, intervention with anti-inflammatory properties and the ability to restore CNS energetic dysfunction may be a reasonable option for fatigue treatment.

Ketogenic diet (KD) is a nutritional strategy initially introduced by Russell Wilder in 1920 as a non-drug treatment for epilepsy. He also coined the term “ketogenic diet” at that time [19,20]. This diet gained popularity in the 1970s, emerging as a potential therapy for various health conditions and as an effective short-term approach to weight loss [21,22]. By drastically reducing carbohydrate intake and increasing the consumption of fats and proteins, this dietary approach induces a metabolic state known as “ketosis”, where fats become the primary energy source instead of carbohydrates.

Recent research highlights the potential benefits of the ketogenic diet in lowering the risk of certain diseases, including type 2 diabetes, hyperlipidemia, cardiovascular conditions, and cancer [23]. The ketogenic diet is characterized by a high intake of fats, a moderate amount of protein, and a low consumption of carbohydrates. Typically, the macronutrient composition includes fats making up 60–90% of the total energy intake (commonly 70–75%), carbohydrates limited to less than 50 g per day (about 5–10% of total caloric intake), and proteins ranging from 1.0 to 1.2 to 1.7 g per kilogram of body weight (accounting for around 20% of daily caloric intake) [24].

The primary objective of all ketogenic diets (KDs) is to stimulate ketone production while ensuring that the body receives adequate caloric intake. KDs exhibit notable anti-inflammatory properties through various molecular pathways and have shown promising results in the management of neurological conditions in which inflammation plays a key role [25].

Additionally, ketone bodies generated during this diet serve as a highly efficient energy source, significantly increasing the ATP/ADP ratio in the brain [9,14]. Ketone bodies are also a preferred energy substrate for oligodendrocytes, supporting energy production and myelin synthesis [26,27].

The anti-inflammatory properties of KD have been documented across several disease contexts. In drug-resistant epilepsy, KD reduces neuroinflammation by inhibiting the NLRP3 inflammasome and NF-κB signaling pathways, with reductions in pro-inflammatory cytokines including IL-1β, IL-6, and TNF-α. In type 2 diabetes and metabolic syndrome, multiple randomized controlled trials have documented reductions in IL-6 and other inflammatory markers with KD compared to low-fat diets [25]. In obesity, KD is associated with reductions in adipose tissue inflammation and circulating inflammatory mediators. In neurodegenerative conditions—including Alzheimer’s disease and Parkinson’s disease—KD and ketone supplementation have shown anti-inflammatory effects in both animal models and pilot clinical studies [28,29]. In MS specifically, a murine EAE model study demonstrated that KD attenuated neuroinflammation and promoted a shift from pro-inflammatory M1 to anti-inflammatory M2 microglia phenotype through NF-κB/NLRP3 and HDAC3/P2X7R pathway modulation [30]. These converging lines of evidence support the biological plausibility of KD as an anti-inflammatory intervention in MS.

Furthermore, the effect of KD on sleep quality and daytime somnolence has been examined in an exploratory study of patients affected by multiple sclerosis [31].

Building on this theoretical foundation, the present study aimed to assess the effects of KD on fatigue and other symptoms, such as sleepiness and sleep disturbances, in a carefully selected group of individuals with MS.

## 2. Materials and Methods

### 2.1. Study Design and Participants

This was a prospective, single-center, non-pharmacological interventional study conducted in volunteers.

**Figure 1:**
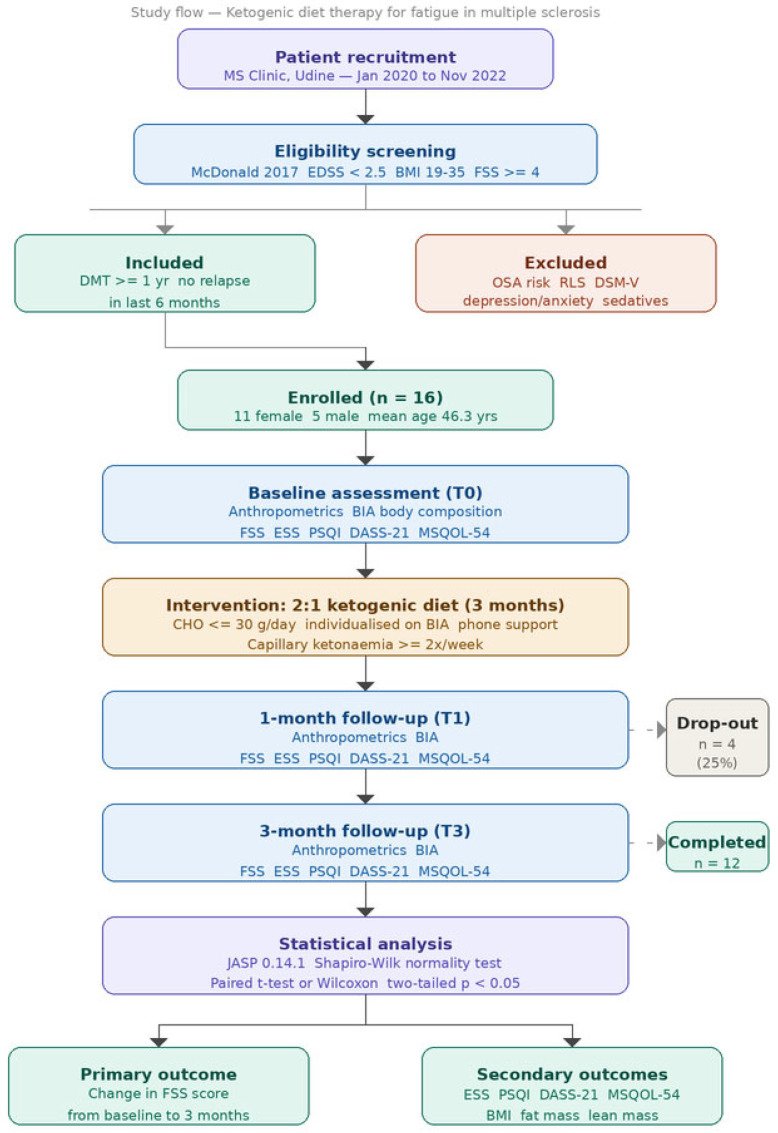
Study flow diagram. All consecutive patients attending the MS clinic between January 2020 and November 2022 who met the eligibility criteria were screened and offered participation. Reasons for non-enrolment and drop-out are specified. T0 = baseline; T1 = 1-month follow-up; T3 = 3-month follow-up; BIA = bioelectrical impedance analysis; FSS = Fatigue Severity Scale; ESS = Epworth Sleepiness Scale; PSQI = Pittsburgh Sleep Quality Index; DASS-21 = Depression Anxiety Stress Scales-21; MSQOL-54 = Multiple Sclerosis Quality of Life-54.

From January 2020 to November 2022, we selected and enrolled patients who attended our multiple sclerosis and demyelinating diseases clinic (Clinical Neurology Unit, S. Maria della Misericordia University Hospital, Udine, Italy). All consecutive patients attending our MS clinic who met the eligibility criteria were screened and offered participation in the study. Patients who declined participation despite fulfilling all inclusion criteria were not enrolled; their number and the primary reason for declining participation (e.g., insufficient motivation, inability to commit to the dietary restrictions, or logistic constraints) are reported in the study flow diagram (Figure 1).

The complete study flow, from screening to final analysis, is illustrated in Figure 1.

### 2.2. Inclusion and Exclusion Criteria

Participants were individuals with relapsing–remitting multiple sclerosis (RRMS) who were non-disabled or minimally disabled according to the 2017 McDonald criteria [32], who were treated with disease-modifying therapies (DMTs) for at least one year, and had no clinical or neuroradiological relapses in the six months prior to or during the study. Additionally, participants had no significant contraindications to the ketogenic diet (KD).

We excluded patients with conditions that could interfere with sleep and fatigue in individuals with MS, including those at intermediate or high risk for obstructive sleep apnea (OSA) as assessed by the STOP-Bang questionnaire [33], those who met the international diagnostic criteria for restless legs syndrome (RLS) [34], and patients diagnosed with depressive or anxiety disorders according to the DSM-V criteria [35,36]. Furthermore, patients taking antidepressants, benzodiazepines, or other sedative medications were excluded from the study (Table 1).

### 2.3. Ethical Aspects

Patients were provided with comprehensive written information about the study, including details on the study design, expected benefits, and potential adverse events associated with the ketogenic diet (KD). All patients provided written informed consent to participate in the study. The study was conducted in accordance with the Declaration of Helsinki and approved by the Comitato Etico Unico Regionale del Friuli Venezia Giulia (CEUR-2020-SPER-124).

### 2.4. Dietetic Intervention and Nutritional Evaluations

Each patient underwent an initial nutritional assessment at enrolment, during which height, weight, and Body Mass Index (BMI) were recorded. Body composition was assessed using bioelectrical impedance analysis with the BIA 101 BIVA PRO (Akern, Pisa, Italy) device. Based on these data and the individual characteristics of each patient, a 2:1 ketogenic diet was prescribed, which patients were instructed to follow for three months, provided it was well tolerated.

The dietary regimen was individualized according to patients’ preferences and body composition parameters (BMI, fat mass, and fat-free mass). Carbohydrate intake was fixed at 30 g/day as an upper limit, in accordance with published clinical recommendations for ketogenic diets in neurological conditions [24]. This threshold has been shown to reliably induce nutritional ketosis (capillary blood beta-hydroxybutyrate ≥ 0.5 mmol/L) in the majority of individuals. While the carbohydrate limit represented a fixed constraint, all other macronutrients were fully individualized: protein intake was calculated primarily based on fat-free mass (FFM), adjusted for physical activity level and ideal body weight; fat intake was determined accordingly to meet individualized caloric requirements derived from anthropometric and body composition data.

Patients were re-evaluated after 1 month (T1) and 3 months (T3) from diet initiation. During follow-up visits, anthropometric and body composition measurements were repeated, and practical advice was provided to improve dietary implementation.

Adherence to the ketogenic regimen was assessed through a combination of objective and subjective measures. As the primary objective indicator, patients were provided with a portable ketone meter (Freestyle Optium Neo, Abbott Diabetes Care, Dublin, Ireland) and instructed to measure capillary blood beta-hydroxybutyrate (BHB) levels at home at least twice per week, preferably in the morning in a fasted state, following specific training provided by the study dietitian at the initial visit. Nutritional ketosis was defined as a capillary BHB level ≥ 0.5 mmol/L.

Results were recorded in a dedicated diary and reviewed by the study team at each follow-up visit. As a semi-objective measure, a structured dietary recall was performed by the study dietitian at T1 and T3 to identify systematic non-compliance. Body composition changes—specifically reductions in fat mass with lean mass preservation—served as an additional indirect marker of metabolic adherence. Finally, patients self-reported their overall adherence level and any difficulties encountered at each visit; telephone support was available throughout the study period.

Ketone measurements were interpreted considering the known physiological variability of ketone body kinetics during KD adaptation, including potential temporal changes related to increased peripheral utilization. Therefore, adherence was defined based on a threshold value (BHB ≥ 0.5 mmol/L) and supported by repeated measurements over time rather than single time-point assessments. The use of capillary blood BHB, rather than urinary ketones, provided a more reliable and quantitative measure of the ketotic state throughout the intervention [37].

### 2.5. Outcomes

Clinical and demographic characteristics were collected at baseline. Data regarding adverse events or relapses were collected during follow-up by means of monthly phone calls and subsequent medical examination, if deemed necessary by study investigators.

The primary objective of the study was to assess the change in fatigue severity from baseline to 3 months, as measured by the Fatigue Severity Scale, in a group of patients with relapsing–remitting multiple sclerosis undergoing a ketogenic diet.

Secondary objectives were to evaluate, in the same patient population, the differences between baseline and the 3-month follow-up with respect to the following aspects: sleep quality, mood disturbances and quality of life. Thus, at baseline and 1 and 3 months after diet initiation, the following questionnaires were administered, all in their validated Italian version: Fatigue Severity Scale (FSS) [7,38], Epworth Sleepiness Scale (ESS) [39,40], Pittsburgh Sleep Quality Index (PSQI) [41,42], Depression Anxiety Stress Scales-21 (DASS-21) [43,44], Multiple Sclerosis Quality of Life-54 (MSQOL-54) [45,46]. All the validated Italian versions of the questionnaires used in the study are included in the Supplementary Materials.

### 2.6. Fatigue Severity Scale (FSS)

Fatigue is a symptom assessed through self-reporting. The Fatigue Severity Scale (FSS) is a 9-item tool that evaluates the average level of fatigue experienced by patients in the previous days. It gauges both the intensity of fatigue and its impact on a person’s daily activities and quality of life under various conditions. Originally developed for multiple sclerosis and systemic lupus erythematosus, it has also been applied to migraines [47]. This scale assesses how fatigue in chronic conditions affects cognitive and physical functioning. Patients rate the severity of their fatigue symptoms for each item, with lower scores indicating disagreement and higher scores indicating agreement. Each statement is scored on a 7-point scale, from 1 (strongly disagree) to 7 (strongly agree). The total score is calculated by summing the individual responses and dividing by nine, although some studies use the total sum score instead. A score of at least 4 (or 36) is considered indicative of pathological fatigue [48].

### 2.7. Pittsburgh Sleep Quality Index (PSQI) and Epworth Sleepiness Scale (ESS)

The Epworth Sleepiness Scale (ESS) is a questionnaire widely used worldwide to subjectively assess the tendency to fall asleep in specific daytime situations, such as reading, watching TV, or being a passenger in a car.

The ESS is an 8-item test developed by Murray J. in 1991 [39]. It is self-administered and provides a score on a numerical scale ranging from 0 to 24. A score greater than 10 is generally accepted as indicative of Excessive Daytime Sleepiness.

The PSQI (Pittsburgh Sleep Quality Index) is a rating scale designed to provide a reliable, valid, and standardized measure of sleep quality. The scale consists of 19 items self-assessed by an individual. These 19 items are grouped into seven composite components that represent subjective sleep quality, sleep latency, sleep duration, habitual sleep efficiency, sleep disturbances, use of hypnotic medication, and daytime dysfunction [49].

### 2.8. Data and Statistical Analysis

Statistical analysis was performed using JASP version 0.14.1 for macOS (University of Amsterdam, The Netherlands). Categorical variables were reported as percentages; continuous variables were reported as mean ± standard deviation. The normality of continuous variables was assessed using the Shapiro–Wilk test, which guided the choice of the appropriate statistical tests. Descriptive analyses were conducted to report means and standard deviations. Comparisons between baseline and subsequent time points were performed using the paired-samples *t*-test or the Wilcoxon signed-rank test, as appropriate. The paired-samples *t*-test was applied to normally distributed variables, whereas non-normally distributed variables were analyzed using the Wilcoxon signed-rank test. The specific test used for each variable is indicated in the corresponding table footnotes. All tests were two-tailed, and *p*-values < 0.05 were considered statistically significant.

## 3. Results

Sixteen patients were enrolled in this study at the end of February 2023.

Eleven (68.75%) patients were females, and five (31.25%) were males. Mean age was 46.31 ± 10.63 years, and mean time from MS diagnosis was 10.31 ± 7.14 years. All patients had a basal EDSS score between 1 and 2. The DMDs distribution was as follows: seven (43.75%) dimethyl fumarate, three (18.75%) teriflunomide, three (18.75%) glatiramer acetate, and three (18.75%) interferons (Table 2).

Four patients did not complete the 3-month study period. One patient stopped the study because of difficulties in following the diet due to the necessity of frequently not eating at home for work reasons, and one found the diet too restrictive. Two patients abandoned the diet due to side effects: one patient experienced weight loss that was perceived as excessive by the patient, but with a BMI still in the range of normality, while another reported abdominal pain soon after diet initiation.

In addition to the cases that dropped due to excessive weight loss and abdominal pain, other adverse events reported by the patients included: dermatitis, observed in one patient, which resolved in a few days and was considered by the investigators not related to the diet; and muscle cramps, observed at the third month of diet in one patient who was not correctly assuming the prescribed food supplement and resolved after it was correctly assumed.

None of the patients experienced an MS relapse during the 3 months of diet.

**Figure 2:**
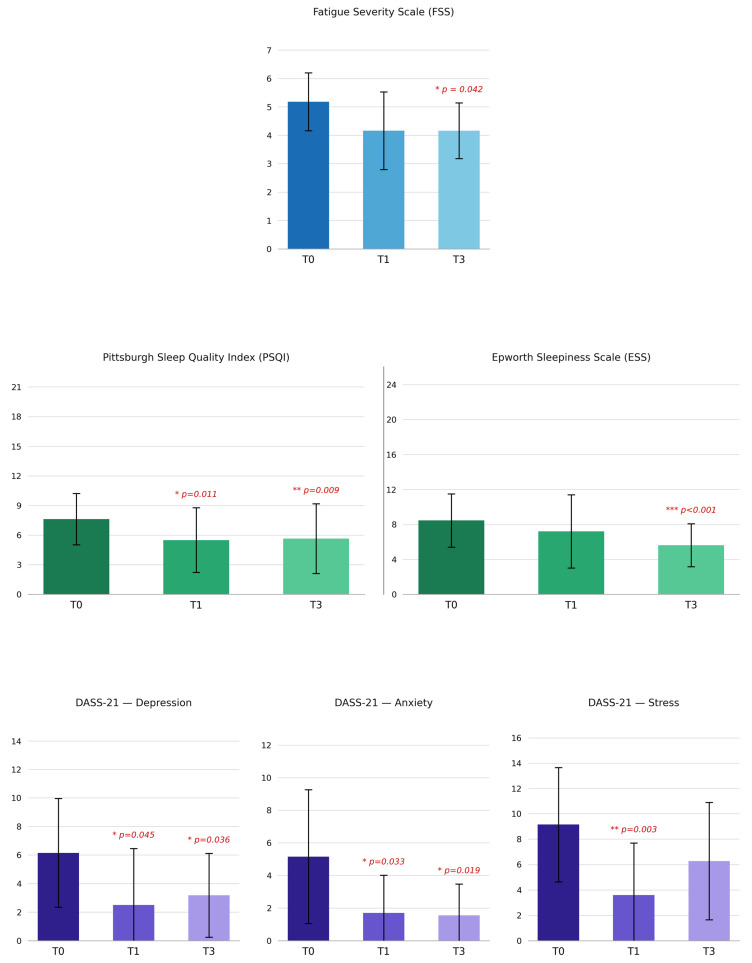
Results of questionnaires at baseline and after one and three months of KD. Data are mean ± SD. * *p* < 0.05, ** *p* < 0.01, *** *p* < 0.001 vs. baseline. The *p*-values are from paired-samples *t*-test for normally distributed variables (assessed by Shapiro–Wilk test) or Wilcoxon signed-rank test for non-normally distributed variables, as appropriate. FSS: Fatigue Severity Scale; ESS: Epworth Sleepiness Scale; PSQI: Pittsburgh Sleep Quality Index; DASS 21: Depression Anxiety Stress Scales-21.

Considering our primary outcome of fatigue, we observed a trend for reduction in FSS scores after 1 month of diet (4.16 ± 1.37 vs. 5.18 ± 1.02; *p* = 0.052), which became significant at 3 months (4.16 ± 0.98 vs. 5.18 ± 1.02; *p* = 0.042). Considering the other outcome measures, ESS scores also improved, reaching a statistically significant reduction at 3 months. The PSQI and the subscales for depression and anxiety of the DASS-21 all showed a better score during KD with a significant reduction at both 1 and 3 months. The stress subscale of the DASS-21 showed an initial improvement which was not consistent at 3 months. Table 3 shows the results of the fatigue, sleep and mood scales during the diet (Figure 2).

Despite a trend toward better scores, we did not observe any significant modification of the physical health composite score or mental health composite scores of the MSQOL-54. The only MSQOL-54 subscale that showed a significant change from the baseline was that of energy at 3 months (37.43 ± 14.26 at baseline, 52.49 ± 12.83 at three months, *p* = 0.042). Table 4 shows the MSQOL-54 subscales variations during the study period.

From a nutritional point of view, we observed a significant reduction in Body Mass Index (BMI) and fat mass (FM) at both 1 and 3 months, while lean mass was preserved at 1 month and even increased after 3 months of KD (Table 5).

## 4. Discussion

In the present study, 4 of 16 patients (25%) discontinued the diet because of tolerability issues. Globally, these are good adherence rates, since in the setting of epilepsy, a 2014 review that considered only adults found a drop-out rate of 51% when considering classic KDs and of 42% when considering a modified Atkins diet [50]. In our experience, in a group of patients affected by migraine, treated with a KD with the same ketogenic ratio, we observed a drop-out rate of 18%, which is quite similar to the findings of the present study [51], thus not suggesting specific limitations of application of this approach in the MS population.

All the side effects observed in our study were transient, and KD was discontinued because of adverse events only in two cases. Muscle cramps and excessive weight loss are frequent side effects of KD, and abdominal pain has also been described, although more rarely [24]. The dermatitis reported was not deemed related to KD by the study investigators.

We did not observe any clinical relapse of MS during the study period. In the previous studies using ketogenic diet in MS, Choi et al. reported only one relapse in their group of 20 patients, while Brenton et al. did not observe relapses in their group of 56 patients [52,53]. Both studies lasted for 6 months. The small number of patients considered in these studies and in our study, and the short time of observation, make it impossible to draw any conclusions about diet efficacy in reducing the relapse rate in MS.

Considering our primary outcome measure, we observed a reduction in fatigue as measured by FSS, which was statistically significant after 3 months of diet. Similarly, we observed an improvement at 3 months in the energy subscale of the MSQOL-54. To our knowledge, two studies by the same American group have evaluated the effect of KD on MS symptoms, with similar positive results [52,54]. However, since in both studies the majority of the sample was formed by overweight or obese patients [52,54], and given that higher BMI has been associated with higher fatigue scores both in pwMS [55] and in people not suffering from this disease [56], the findings of these studies must be taken with caution. It must also be noted that in those studies, the dietary intervention was relatively simple: subjects were only instructed to restrict carbohydrates to <20 g/day without further macronutrient individualization [52]. By contrast, our protocol prescribed a fixed carbohydrate ceiling of 30 g/day within a fully individualized 2:1 ketogenic regimen in which protein and fat intakes were calculated for each patient based on body composition parameters and physical activity level. Furthermore, the results of our study, in which patients with significant obesity were excluded (BMI upper limit 35 kg/m^2^) and the mean BMI of the enrolled sample was 23.54 ± 2.98 kg/m^2^—substantially lower than that of the predominantly overweight or obese samples in the aforementioned studies—offer more robust evidence of the usefulness of KD in reducing fatigue in pwMS with a lower degree of weight-related confounding.

We also observed a consistent reduction in excessive daytime sleepiness as measured by ESS, and an improvement in sleep quality as measured by PSQI. To our knowledge, this is the first study to assess sleep in patients with pwMS receiving KD, although sleep disturbances are a common problem in the MS population [31,57]. In addition, considering the application of KD in other settings, data about sleep and sleepiness remain scarce [58,59].

Several mechanisms by which KD may influence sleep architecture and daytime somnolence have been proposed. Relative hypoglycemia induced by carbohydrate restriction may activate orexinergic neurons, promoting wakefulness consolidation and reducing excessive daytime somnolence. Increased galanin expression under KD conditions may inhibit the monoaminergic arousal system, facilitating deeper sleep. Additionally, KD modulates the GABA/glutamate ratio—increasing inhibitory GABAergic tone—which is thought to promote sleep initiation and continuity [60,61,62,63]. The reduction in systemic neuroinflammation may further contribute by attenuating cytokine-driven disruption of normal sleep–wake regulation.

In our study, we found that patients with MS and KD showed a significant reduction in depression and anxiety scores. Considering KD studies in patients with MS, the only other studies that evaluated the effect of this dietary approach on mood disturbances are those already mentioned by Brenton et al. [52,54], in which pwMS receiving a KD reported a reduction in Beck Depression Inventory scores. In addition to MS, KD has been shown to improve negative affect also in overweight but otherwise healthy patients [64] and in a mixed cohort of psychiatric inpatients [65]. KD application in the treatment of depression and anxiety is also supported by animal data [66,67]. The mechanism by which KD ameliorates depression and anxiety is not clear, but modulation of GABAergic and monoaminergic systems may be involved, along with chronic low-grade inflammation reduction and mitochondrial function restoration [68,69]. At the cellular level, the modulation of GABAergic tone by KD may reduce neuronal hyperexcitability associated with anxiety states [60], while an increase in brain-derived neurotrophic factor (BDNF) reported in some KD studies may support neuroplasticity pathways implicated in mood regulation [70,71]. The reduction in systemic low-grade inflammation—particularly of IL-6 and TNF-α—is also relevant, given the established role of the inflammatory–depression axis in MS [72]. Our finding of a reduction in depression and anxiety symptoms in pwMS receiving a KD are relevant in relation to the extent of the phenomenon, since depression and anxiety prevalence among pwMS can be as high as 30.5% and 22.1% respectively [73], and depression, together with fatigue and disability, is one of the most important determinants of quality of life in these patients [74].

Although an in-depth description of the effect of KD on weight and fat mass reduction is beyond the scope of this study, we have at least to comment that the observed reduction in BMI and FM with lean mass preservation corroborates the already known possibility of KD utilization for weight loss [22,75,76,77]. As previously discussed, we did not include obese patients in this study, and the median BMI of the study group was within the normal range before and after the dietary intervention.

Potential sex- and age-related differences in the metabolic and clinical response to KD could not be formally explored in the present study given the limited sample size, although the cohort was predominantly female (68.75%), consistent with the known sex distribution of RRMS. These variables should be specifically addressed in future larger studies.

A further limitation concerns the restricted range of neurological disability and disease phase in our sample. All enrolled patients had EDSS scores between 1 and 2 and were in the relapsing–remitting phase of MS. This selection was intentional—aimed at minimizing confounding from disability-related deconditioning and secondary fatigue—but it limits the generalizability of our findings to patients with more advanced or progressive MS, in whom the pathophysiological substrate of fatigue (greater axonal loss, more extensive neurodegeneration, and reduced treatment responsiveness) may differ substantially. With respect to disease duration, the mean time from diagnosis in our sample was 10.31 ± 7.14 years, indicating considerable heterogeneity. Whether the anti-inflammatory and metabolic benefits of KD are modulated by disease stage, duration, or phase remains an open question that future studies should specifically address, ideally enrolling patients across a broader disability spectrum.

A major limitation of this study is the absence of a control or comparator dietary arm. Without a control group, it is not possible to exclude contributions from non-specific effects such as increased dietary attention, the Hawthorne effect, regression to the mean, or natural fluctuation in MS symptom severity over time. The results should therefore be interpreted as preliminary and hypothesis-generating, requiring confirmation in a randomized controlled trial comparing KD against a standardized healthy diet control.

It must also be emphasized that the ketogenic diet is not without challenges and is not universally applicable or beneficial. As noted above, 25% of patients in our study discontinued the intervention before the 3-month endpoint, due to either adverse events or difficulties maintaining dietary restrictions in everyday life—rates consistent with or lower than those reported for KD in adult epilepsy [78]. Sustaining a KD requires a high degree of motivation, careful food planning, habitual nutritional label reading, avoidance of most traditional carbohydrate-containing foods, and regular self-monitoring of ketone levels. Social situations involving shared meals, travel, and festive occasions present particular adherence challenges. The initial adaptation phase may also be accompanied by transient symptoms—collectively termed ‘keto flu’—including fatigue, headache, nausea, and irritability, which may paradoxically discourage continuation in a population already burdened by fatigue [79,80]. Long-term sustainability is a key concern in a chronic disease context such as MS, where any nutritional intervention would need to be maintained over years rather than months. Furthermore, although individual variability in the degree and timing of ketosis induction was present in our study, the use of capillary blood BHB monitoring—rather than urinary ketone measurement, which is less reliable at later stages of keto-adaptation—provided a more objective framework for adherence assessment. The lack of systematic retention of ketone data for quantitative analysis represents a further limitation, as it precludes a formal assessment of the relationship between the depth of ketosis achieved and the observed clinical outcomes. Future studies should include systematic collection and reporting of ketone body levels as a continuous variable.

Finally, KD is contraindicated in patients with renal insufficiency, hepatic disease, significant dyslipidemia, eating disorders, porphyria, or pyruvate carboxylase deficiency, and requires caution in patients receiving medications that interact with carbohydrate metabolism. Appropriate patient selection and multidisciplinary supervision—involving at minimum a neurologist experienced in MS management and a qualified dietitian—are essential prerequisites for safe and effective implementation of this dietary intervention [81].

## 5. Conclusions

After the great results reached in the prevention of relapses and disability worsening in MS with the introduction of many disease-modifying drugs, the research focused on treating more subtle, but still very disabling symptoms, such as fatigue. Although the exact processes behind this symptom remain partially unclear, persistent inflammation and neuronal energetic dysfunction are suggested to be involved [82,83,84]. In this study, we aimed to evaluate the application of KD in a group of RRMS patients, given the anti-inflammatory and energetic properties of this diet [24].

First, we found that ketogenic intervention is applicable to MS patients with limitations similar to those of other dietary interventions and with a higher adherence than that described for KD in other disorders.

Regarding our primary outcome, we observed an improvement in fatigue during KD. In parallel, KD resulted in the significant amelioration of sleep and daytime somnolence, and a reduction in depression and anxiety symptoms.

We are aware that the present study has several limitations. First, the sample size was limited and, given the chronic nature of MS, longer observation times are required to assess long-term efficacy and tolerability of KD. Another limitation is the lack of a control group. The use of laboratory and instrumental outcomes may also help corroborate KD utilization in this field, and studies including these targets are ongoing at our center.

The present study also has some strengths, dependent on the accuracy of the sample selection, which gave us the possibility of excluding many confounding factors, and on the precise diet prescriptions adopted.

Further studies with longer follow-up periods, larger sample sizes and a control group are needed to firmly establish KD efficacy on MS symptoms and course.

## Figures and Tables

**Table 1:** Inclusion and exclusion criteria for study participation.

Inclusion Criteria	Exclusion Criteria
Diagnosis of (RR)MS following revised McDonald criteria (CIT) Age between 18 and 65 years EDSS < 2.5 at enrolment Patients on DMD for at least 1 year In treatment with one of the following DMDs: glatimer acetate, teriflunomide, dimethyl fumarate, interferons Clinically relevant fatigue (FSS ≥ 4) BMI between 19 and 35 kg/m^2^	Renal failure (estimated glomerular filtration rate with Cockroft-Gault formula < 60 mL/min) History of urinary stone Hepatic failure Known cardiopathies History of arrhythmia or conduction abnormalities on baseline electrocardiogram (not including right branch block and type I atrio-ventricular block) Diabetes mellitus Porphyria Known deficit of pyruvate carboxylase Known disorders of lipid metabolism Ischemic stroke or transient ischemic attack in the previous 6 months Pregnancy and lactation History of acute or chronic pancreatitis Severe osteoporosis Known thyroid dysfunction Alcohol abuse Eating disorders Diagnosis of epilepsy or seizure in the past 2 years Diagnosis of major depressive disorder Treatment with antidepressants, benzodiazepines or hypnotic drugs Relapses of MS in the last 6 months Steroid treatment in the last 6 months New MRI demyelinating lesions in the previous 6 months

**Table 2:** Baseline characteristics of all enrolled patients. Data are mean ± SD unless otherwise stated. No formal statistical comparison between completers and drop-outs is reported: with only four individuals in the drop-out group, any test would be insufficiently powered to distinguish a true difference from random variation. Descriptive inspection reveals no clinically meaningful differences in baseline characteristics between the two groups. EDSS = Expanded Disability Status Scale; RRMS = Relapsing–Remitting Multiple Sclerosis; BMI = Body Mass Index; FSS = Fatigue Severity Scale; ESS = Epworth Sleepiness Scale; PSQI = Pittsburgh Sleep Quality Index; DASS-21 = Depression Anxiety Stress Scales-21; MSQOL-54 = Multiple Sclerosis Quality of Life-54.

Characteristic	All Enrolled (*n* = 16)	Completers (*n* = 12)	Drop-Outs (*n* = 4)
***Demographics***			
Age, years (mean ± SD)	46.31 ± 10.63	47.08 ± 10.94	43.75 ± 10.53
Sex			
Female—*n* (%)	11 (68.8%)	8 (66.7%)	3 (75.0%)
Male—*n* (%)	5 (31.3%)	4 (33.3%)	1 (25.0%)
Disease duration, years (mean ± SD)	10.31 ± 7.14	10.75 ± 7.54	9.00 ± 5.72
***Neurological characteristics***			
EDSS at baseline (range)	1.0–2.0	1.0–2.0	1.0–2.0
MS phenotype	RRMS (100%)	RRMS (100%)	RRMS (100%)
***Disease-modifying therapy (n, %)***			
Dimethyl fumarate	7 (43.8%)	5 (41.7%)	2 (50.0%)
Teriflunomide	3 (18.8%)	2 (16.7%)	1 (25.0%)
Glatiramer acetate	3 (18.8%)	3 (25.0%)	0 (0.0%)
Interferons	3 (18.8%)	2 (16.7%)	1 (25.0%)
***Anthropometric and body composition measures (mean ± SD)***			
BMI, kg/m^2^	23.54 ± 2.98	23.41 ± 3.11	24.00 ± 2.45
Fat mass, kg	18.73 ± 8.26	18.50 ± 8.63	19.50 ± 7.18
Lean mass, kg	49.52 ± 8.37	49.80 ± 8.74	48.75 ± 7.35
Intracellular water, L	18.99 ± 4.42	19.10 ± 4.55	18.60 ± 4.18
Extracellular water, L	17.33 ± 2.05	17.40 ± 2.10	17.10 ± 1.95
***Baseline clinical scale scores (mean ± SD)***			
FSS	5.18 ± 1.02	5.24 ± 1.07	5.00 ± 0.94
ESS	8.46 ± 3.05	8.58 ± 3.18	8.00 ± 2.71
PSQI	7.62 ± 2.59	7.75 ± 2.67	7.25 ± 2.36
DASS-21			
- depression subscale	6.15 ± 3.81	6.33 ± 4.01	5.50 ± 3.32
- anxiety subscale	5.15 ± 4.10	5.33 ± 4.29	4.50 ± 3.70
- stress subscale	9.15 ± 4.51	9.33 ± 4.73	8.50 ± 4.04
MSQOL-54			
- physical composite score	60.87 ± 12.91	60.42 ± 13.54	62.00 ± 11.18
- mental composite score	59.90 ± 15.35	59.33 ± 16.01	62.00 ± 13.54
- energy subscale	37.43 ± 14.26	37.20 ± 14.80	38.25 ± 13.50

**Table 3:** Results of questionnaires at baseline and after one and three months of KD. Data are mean ± SD. * *p* < 0.05 vs. baseline. The *p*-values are from paired-samples *t*-test for normally distributed variables (assessed by Shapiro–Wilk test) or Wilcoxon signed-rank test for non-normally distributed variables, as appropriate. FSS: Fatigue Severity Scale; ESS: Epworth Sleepiness Scale; PSQI: Pittsburgh Sleep Quality Index; DASS 21: Depression Anxiety Stress Scales-21.

	Baseline (Mean ± SD)	1 Month (Mean ± SD)	3 Months (Mean ± SD)
FSS	5.18 ± 1.02	4.16 ± 1.37; *p* = 0.052	4.16 ± 0.98; *p* = 0.042 *
ESS	8.46 ± 3.05	7.20 ± 4.19; *p* = 0.127	5.64 ± 2.46; *p* < 0.001 *
PSQI	7.62 ± 2.59	5.50 ± 3.27; *p* = 0.011 *	5.64 ± 3.53; *p* = 0.009 *
DASS-21 depression	6.15 ± 3.81	2.50 ± 3.95; *p* = 0.045 *	3.18 ± 2.93; *p* = 0.036 *
DASS-21 anxiety	5.15 ± 4.10	1.70 ± 2.31; *p* = 0.033 *	1.55 ± 1.92; *p* = 0.019 *
DASS-21 stress	9.15 ± 4.51	3.60 ± 4.09; *p* = 0.003 *	6.273 ± 4.63; *p* = 0.208

**Table 4:** MSQOL-54 subscales scores at baseline and after different months of KD. Data are mean ± SD. * *p* < 0.05 vs. baseline. The *p*-values are from paired-samples *t*-test for normally distributed variables (assessed by Shapiro–Wilk test) or Wilcoxon signed-rank test for non-normally distributed variables, as appropriate.

	Baseline (Mean ± SD)	1 Month (Mean ± SD)	3 Months (Mean ± SD)
Physical health	78.57 ± 16.81	85.46 ± 10.59; *p* = 0.057	84.09 ± 13.38; *p* = 0.146
Role limitations due to physical problems	41.07 ± 38.74	77.27 ± 28.41; *p* = 0.148	72.73 ± 34.38; *p* = 0.138
Role limitations due to emotional problems	50.00 ± 48.48	75.76 ± 36.79; *p* = 0.191	66.67 ± 42.16; *p* = 0.893
Pain	70.64 ± 22.31	80.30 ± 18.47; *p* = 0.400	84.85 ± 19.73; *p* = 0.172
Emotional well-being	58.57 ± 16.12	69.46 ± 17.28; *p* = 0.128	67.09 ± 16.13; *p* = 0.314
Energy	37.43 ± 14.26	50.90 ± 17.54; *p* = 0.056	52.49 ± 12.83; *p* = 0.042 *
Health perceptions	45.71 ± 17.08	53.18 ± 13.65; *p* = 0.271	52.78 ± 14.73; *p* = 0.276
Social function	66.67 ± 21.68	82.58 ± 15.57; *p* = 0.072	82.58 ± 16.86; *p* = 0.288
Cognitive function	66.07 ± 18.42	72.27 ± 12.12; *p* = 0.125	75.00 ± 15.65; *p* = 0.120
Health distress	73.21 ± 15.01	78.64 ± 12.67; *p* = 0.916	78.18 ± 13.83; *p* = 0.945
Sexual function	69.23 ± 30.13	64.82 ± 33.02; *p* = 0.732	75.00 ± 28.87; *p* = 0.138
Change in health	44.64 ± 20.05	56.82 ± 22.61; *p* = 0.414	61.36 ± 23.36; *p* = 0.177
Satisfaction with sexual function	53.57 ± 32.31	50.00 ± 35.36; *p* = 1.000	56.82 ± 33.71; *p* = 0.726
Overall quality of life	63.33 ± 16.27	69.88 ± 12.97; *p* = 0.629	72.39 ± 13.76; *p* = 0.207
Physical health composite score	60.87 ± 12.91	73.32 ± 11.34; *p* = 0.316	72.18 ± 13.22; *p* = 0.178
Mental health composite score	59.90 ± 15.35	72.75 ± 14.95; *p* = 0.130	69.77 ± 17.72; *p* = 0.770

**Table 5:** Nutritional data at baseline and after 1 month of KD. Data are mean ± SD. * *p* < 0.05 vs. baseline. The *p*-values are from paired-samples *t*-test for normally distributed variables (assessed by Shapiro–Wilk test) or Wilcoxon signed-rank test for non-normally distributed variables, as appropriate.

	**Baseline** **(Mean ± SD)**	**1 Month** **(Mean ± SD)**	**3 Months** **(Mean ± SD)**
Body Mass Index (BMI) (kg/m^2^)	23.54 ± 2.98	22.56 ± 2.49; *p* = 0.004 *	23.26 ± 2.04; *p* = 0.004 *
Fat mass (kg)	18.73 ± 8.26	16.31 ± 7.70; *p* = 0.002 *	17.43 ± 7.70; *p* = 0.034 *
Lean mass (kg)	49.52 ± 8.37	47.67 ± 6.24; *p* = 0.064	49.94 ± 8.43; *p* = 0.010 *
Intracellular water (L)	18.99 ± 4.42	18.87 ± 4.65; *p* = 0.248	19.30 ± 4.25; *p* = 0.059
Extracellular water (L)	17.33 ± 2.05	17.21 ± 5.58; *p* = 0.170	17.31 ± 2.22; *p* = 0.014 *

## Data Availability

The original contributions presented in this study are included in the article/Supplementary Materials. Further inquiries can be directed to the corresponding author.

## Supplementary Materials

The following supporting information can be downloaded at: https://www.mdpi.com/article/10.3390/nu18101496/s1. The validated Italian versions of all questionnaires administered in the study are provided: Table S1: Fatigue Severity Scale (FSS); Table S2: Epworth Sleepiness Scale (ESS); Table S3: Pittsburgh Sleep Quality Index (PSQI); Table S4: Depression Anxiety Stress Scales-21 (DASS-21); Table S5: Multiple Sclerosis Quality of Life-54 (MSQOL-54). Note: instruments are presented in Italian as this is the language of the validated versions used with study participants.

## Abbreviations

MS	Multiple Sclerosis
KD	Ketogenic Diet
DMTs	Disease-Modifying Therapies
RCTs	Randomized Clinical Trials
pwMS	Patients With Multiple Sclerosis
CNS	Central Nervous System
RRMS	Relapsing–Remitting Multiple Sclerosis
FSS	Fatigue Severity Scale
ESS	Epworth Sleepiness Scale
PSQI	Pittsburgh Sleep Quality Index
DASS-21	Depression Anxiety Stress Scales-21
MSQOL-54	Multiple Sclerosis Quality of Life-54
BMI	Body Mass Index
BIA	Bioelectrical Impedance Analysis
FM	Fat Mass
FFM	Fat Free Mass
