# Supplementary material for: Ketogenic Diet-Based Therapy for Fatigue in Patients with Multiple Sclerosis
Source: Nutrients. 2026 May 8;18(10):1496. doi: 10.3390/nu18101496 (PMC13209564; doi:10.3390/nu18101496)
Supplement: Supplementary file 1 [file nutrients-18-01496-s001.zip › nutrients-4254154-supplementary.pdf]

Fatigue severity scale (FSS)

Il questionario seguente fa riferimento all'ultima settimana. Indichi con un valore da 1 a 7 quanto è in accordo con le seguenti affermazioni (1=per nulla d'accordo; 7=totalmente d'accordo).

La mia motivazione è minore quando sono affaticato	1 2 3 4 5 6 7
L'esercizio mi procura fatica	1 2 3 4 5 6 7
Io sono facilmente affaticato	1 2 3 4 5 6 7
La fatica interferisce con il mio funzionamento fisico	1 2 3 4 5 6 7
La fatica causa frequenti problemi per me	1 2 3 4 5 6 7
La mia fatica impedisce un funzionamento fisico sostenuto	1 2 3 4 5 6 7
La fatica interferisce con lo svolgimento di certi doveri e responsabilità	1 2 3 4 5 6 7
La fatica è tra i miei tre sintomi più disabilitanti	1 2 3 4 5 6 7
La fatica interferisce con il mio lavoro, la famiglia, o la vita sociale	1 2 3 4 5 6 7

Depression Anxiety Stress Scales-21 (DASS 21)

Per favore, legga ogni frase e poi indichi con quale frequenza la situazione descritta si è verificata negli ultimi sette giorni. Esprima la sua valutazione facendo un segno sul numero 0, 1, 2 o 3 secondo la scala di valutazione seguente. Tenga presente che non esistono risposte giuste o sbagliate. Non impieghi troppo tempo per rispondere a ciascuna affermazione, spesso la prima risposta è la più accurata. Grazie per la sua preziosa disponibilità e collaborazione.

	0 Non mi è mai accaduto	1 Mi è capitato qualche volta	2 Mi è capitato con una certa frequenza	3 Mi è capitato quasi sempre
1) Ho provato molta tensione e ho avuto difficoltà a recuperare uno stato di calma	0	1	2	3
2) Mi sono accorto di avere la bocca secca	0	1	2	3
3) Non riuscivo proprio a provare delle emozioni positive	0	1	2	3

4) Mi sono sentito molto in affanno con difficoltà a respirare (per es. respiro molto accelerato, sensazione di forte affanno in assenza di sforzo fisico)	0	1	2	3
5) Ho avuto un'estrema difficoltà nel cominciare quello che dovevo fare	0	1	2	3
6) Ho avuto la tendenza a reagire in maniera 0 eccessiva alle situazioni	0	1	2	3
7) Ho avuto tremori (per es. alle mani)	0	1	2	3
8) Ho sentito che stavo impiegando molta energia nervosa	0	1	2	3
9) Ho temuto di trovarmi in situazioni in cui sarei potuto andare nel panico e rendermi ridicolo	0	1	2	3
10) Non vedevo nulla di buono nel mio futuro	0	1	2	3
11) Mi sono sentito stressato	0	1	2	3
12) Ho avuto difficoltà a rilassarmi	0	1	2	3
13) Mi sono sentito scoraggiato e depresso	0	1	2	3
14) Non riuscivo a tollerare per nulla eventi o situazioni che mi impedivano di portare avanti ciò che stavo facendo	0	1	2	3
15) Ho sentito di essere vicino ad avere un attacco di panico	0	1	2	3
16) Non c'era nulla che mi dava entusiasmo	0	1	2	3
17) Sentivo di valere poco come persona	0	1	2	3
18) Mi sono sentito piuttosto irritabile	0	1	2	3
19) Ho percepito distintamente il battito del mio cuore senza aver fatto uno sforzo fisico (per es. battito cardiaco accelerato o perdita di un battito)	0	1	2	3
20) Mi sono sentito spaventato senza ragione	0	1	2	3
21) Sentivo la vita priva di significato	0	1	2	3

PITTSBURGH SLEEP QUALITY INDEX

<u>LE SEGUENTI DOMANDE SI RIFERISCONO ESCLUSIVAMENTE AL MESE APPENA TRASCORSO</u>

1. A che ora sei andato a dormire di solito?				
2. In media, quanto tempo (in minuti) hai impiegato per addormentarti?				
3. A che ora ti sei svegliato al mattino di solito?				
4. Quante ore hai effettivamente dormito in media (questo valore può essere diverso dal numero di ore trascorse a letto)?				
5. Quanto spesso hai avuto problemi a dormire perché…	Mai durante l'ultimo mese (0)	Meno di una volta a settimana (1)	Una o due volta a settimana (2)	Tre o più volte a settimana (3)
a. Non riuscivi ad addormentarti entro 30 minuti				
b. Ti svegliavi a metà notte o al mattino presto				
c. Dovevi alzarti per andare in bagno				
d. Non riuscivi a respirare bene				
e. Tossivi o russavi rumorosamente				
f. Sentivi troppo freddo				
g. Sentivi troppo caldo				
h. Hai avuto incubi				
i. Avevi dei dolori				
1. Altri motivi: descrivili, indicando quanto spesso hai avuto difficoltà a dormire per queste ragioni				
6. Quanto spesso hai assunto farmaci (prescritti o da banco) per facilitare il sonno?				
7. Quante spesso hai avuto difficoltà a rimanere				

sveglio mentre guidavi, mangiavi o eri impegnato in altre attività?				
8. 8.Quanto spesso hai avuto difficoltà a mantenerti attivo nello svolgere i tuoi compiti quotidiani?				
	Molto buona (0)	Abbastanza buona (1)	Piuttosto scarsa (2)	Molto scarsa (3)
9. Come descriveresti la qualità del tuo sonno?				

Epworth Sleepiness Scale (ESS)

Che probabilità ha di appisolarsi o di addormentarsi nelle seguenti situazioni, indipendentemente dalla sensazione di stanchezza?

La domanda si riferisce alle usuali abitudini di vita nell'ultimo periodo. Qualora non si sia trovato di recente in alcune delle situazioni elencate sotto, provi ad immaginare come si sentirebbe. Usi la seguente scala per scegliere il punteggio più adatto ad ogni situazione:

0 = non mi addormento mai
1 = ho qualche probabilità di addormentarmi
2 = ho una discreta probabilità di addormentarmi
3 = ho un'alta probabilità di addormentarmi

Seduto mentre leggo	
Guardando la TV	
Seduto, inattivo in un luogo pubblico (a teatro, ad una conferenza)	
Passeggero in automobile, per un'ora senza sosta	
Sdraiato per riposare nel pomeriggio, quando ne ho l'occasione	
Seduto mentre parlo con qualcuno	
Seduto tranquillamente dopo pranzo, senza avere bevuto alcoolici	
In automobile, fermo per pochi minuti nel traffico	

MSQOL-54

1998/F

Edizione Italiana

IQOLA SF-36 Italian Version 1.6: Copyright© New England Medical Center Hospitals Inc., 1992. All rights reserved.

Modulo specifico MSQOL-54, versione originale B. Vickrey, 1995; versione Italiana A. Solari, Istituto Nazionale Neurologico C. Besta, Via Celoria 11, 20133 Milano
1998 (1)

ISTRUZIONI: Il questionario intende valutare cosa lei pensa della sua salute. Le informazioni raccolte permetteranno di essere aggiornati su come si sente e su come riesce a svolgere le sue attività' consuete.

Risponda a ciascuna domanda del questionario indicando la sua risposta come mostrato di volta in volta. Se non si sente certa della risposta, effettui la scelta che comunque le sembra migliore.

1. In generale, direbbe che la sua salute è:

(Indichi un numero)

Eccellente 1

Molto buona 2

Buona 3

Passabile 4

Scadente 5

2. <u>Rispetto ad un anno fa</u>, come giudicherebbe, <u>ora</u>, la sua salute in generale?

(Indichi un numero)

Decisamente migliore adesso rispetto ad un anno fa 1

Un po' migliore adesso rispetto ad un anno fa 2

Più o meno uguale rispetto ad un anno fa 3

Un po' peggiore adesso rispetto ad un anno fa 4

Decisamente peggiore adesso rispetto ad un anno fa 5

Le seguenti domande riguardano alcune attività che potrebbe svolgere nel corso di una qualsiasi giornata. La sua salute la limita attualmente nello svolgimento di queste attività?

3. La sua salute la limita attualmente nello svolgimento di **attività fisicamente impegnative**, come correre, sollevare oggetti pesanti, praticare sport faticosi?

(Indichi un numero)

SI, mi limita parecchio 1
SI, mi limita parzialmente 2
NO, non mi limita per nulla 3

4. La sua salute la limita attualmente nello svolgimento di **attività di moderato impegno fisico**, come spostare un tavolo, usare l'aspirapolvere, giocare a bocce o fare un giretto in bicicletta ?

(Indichi un numero)

SI, mi limita parecchio 1
SI, mi limita parzialmente 2
NO, non mi limita per nulla 3

5. La sua salute la limita attualmente nel **sollevare o portare le borse della spesa**?

(Indichi un numero)

SI, mi limita parecchio 1
SI, mi limita parzialmente 2
NO, non mi limita per nulla 3

6. La sua salute la limita attualmente nel **salire qualche piano di scale**?

(Indichi un numero)

SI, mi limita parecchio 1
SI, mi limita parzialmente 2
NO, non mi limita per nulla 3

7. La sua salute la limita attualmente nel **salire un piano di scale**?

(Indichi un numero)

SI, mi limita parecchio 1
SI, mi limita parzialmente 2
NO, non mi limita per nulla 3

8. La sua salute la limita attualmente nel **piegarsi, inginocchiarsi o chinarsi**?

(Indichi un numero)

SI, mi limita parecchio 1
SI, mi limita parzialmente 2
NO, non mi limita per nulla 3

9. La sua salute la limita attualmente nel **camminare per un chilometro**?

(Indichi un numero)

SI, mi limita parecchio 1
SI, mi limita parzialmente 2
NO, non mi limita per nulla 3

10. La sua salute la limita attualmente nel **camminare per qualche centinaia di metri**?

(Indichi un numero)

SI, mi limita parecchio 1
SI, mi limita parzialmente 2
NO, non mi limita per nulla 3

11. La sua salute la limita attualmente nel **camminare per circa cento metri**?

(Indichi un numero)

SI, mi limita parecchio 1
SI, mi limita parzialmente 2
NO, non mi limita per nulla 3

12. La sua salute la limita attualmente nel **fare il bagno o vestirsi da sola**?

(Indichi un numero)

SI, mi limita parecchio 1

SI, mi limita parzialmente 2

NO, non mi limita per nulla 3

Nelle ultime 4 settimane, ha riscontrato i seguenti problemi sul lavoro o nelle altre attività quotidiane, a causa della sua salute fisica?
Risponda SI o NO a ciascuna domanda.

13. Nelle ultime 4 settimane, a causa della sua salute fisica ha ridotto **il tempo dedicato al lavoro o ad altre attività**?

(Indichi per ogni domanda il numero 1 o 2)

SI 1

NO 2

14. Nelle ultime 4 settimane, a causa della sua salute fisica ha reso **meno di quanto avrebbe voluto**?

(Indichi per ogni domanda il numero 1 o 2)

SI 1

NO 2

15. Nelle ultime 4 settimane, a causa della sua salute fisica ha dovuto **limitare alcuni tipi di lavoro o altre attività**?

(Indichi per ogni domanda il numero 1 o 2)

SI 1

NO 2

16. Nelle ultime 4 settimane, a causa della sua salute fisica ha avuto **difficoltà nell'eseguire il lavoro o altre attività** (ad esempio, ha fatto più fatica?)

(Indichi per ogni domanda il numero 1 o 2)

SI 1
NO 2

Nelle ultime 4 settimane, ha riscontrato i seguenti problemi sul lavoro o nelle altre attività quotidiane, a causa del suo stato emotivo (quale il sentirsi depressa o ansiosa)?
Risponda SI o NO a ciascuna domanda.

17. Nelle ultime 4 settimane, a causa del suo stato emotivo ha ridotto il **tempo dedicato al lavoro o ad altre attività**?

(Indichi per ogni domanda il numero 1 o 2)

SI 1
NO 2

18. Nelle ultime 4 settimane, a causa del suo stato emotivo ha **reso meno di quanto avrebbe voluto**?

(Indichi per ogni domanda il numero 1 o 2)

SI 1
NO 2

19. Nelle ultime 4 settimane, a causa del suo stato emotivo ha avuto **un calo di concentrazione** sul lavoro o in altre attività?

(Indichi per ogni domanda il numero 1 o 2)

SI 1
NO 2

20. Nelle ultime 4 settimane, in che misura la sua salute fisica o il suo stato emotivo hanno **interferito con le normali attività sociali** con la famiglia, gli amici, i vicini di casa, i gruppi di cui fa parte?

(Indichi un numero)

Per nulla 1
Leggermente 2
Un po' 3
Molto 4
Moltissimo 5

21. Quanto **dolore fisico** ha provato nelle ultime 4 settimane?

(Indichi un numero)

Nessuno 1
Molto lieve 2
Lieve 3
Moderato 4
Forte 5
Molto forte 6

22. Nelle ultime 4 settimane, in che misura il **dolore l'ha ostacolata** nel lavoro che svolge abitualmente (sia in casa sia fuori casa)?

(Indichi un numero)

Per nulla 1
Molto poco 2
Un po' 3
Molto 4
Moltissimo 5

Le seguenti domande si riferiscono a come si è sentita nelle ultime 4 settimane.
Risponda a ciascuna domanda scegliendo la risposta che più si avvicina al suo caso.

23. Per quanto tempo nelle ultime 4 settimane si è sentita **vivace e brillante**?

(Indichi un numero)

Sempre 1
Quasi sempre 2
Molto tempo 3
Una parte del tempo 4
Quasi mai 5
Mai 6

24. Per quanto tempo nelle ultime 4 settimane si è sentita **molto agitata**?

(Indichi un numero)

Sempre 1
Quasi sempre 2
Molto tempo 3
Una parte del tempo 4
Quasi mai 5
Mai 6

25. Per quanto tempo nelle ultime 4 settimane si è sentita così **giù di morale** che niente avrebbe potuto tirarla su?

(Indichi un numero)

Sempre 1
Quasi sempre 2
Molto tempo 3
Una parte del tempo 4
Quasi mai 5
Mai 6

26. Per quanto tempo nelle ultime 4 settimane si è sentita **calma e serena**?

(Indichi un numero)

Sempre 1
Quasi sempre 2
Molto tempo 3
Una parte del tempo 4
Quasi mai 5
Mai 6

27. Per quanto tempo nelle ultime 4 settimane si è sentita **piena di energia**?

(Indichi un numero)

Sempre 1
Quasi sempre 2
Molto tempo 3
Una parte del tempo 4
Quasi mai 5
Mai 6

28. Per quanto tempo nelle ultime 4 settimane si è sentita **scoraggiata e triste**?

(Indichi un numero)

Sempre 1
Quasi sempre 2
Molto tempo 3
Una parte del tempo 4
Quasi mai 5
Mai 6

29. Per quanto tempo nelle ultime 4 settimane si è sentita **sfinita**?

(Indichi un numero)

Sempre 1
Quasi sempre 2
Molto tempo 3
Una parte del tempo 4
Quasi mai 5
Mai 6

30. Per quanto tempo nelle ultime 4 settimane si è sentita **felice**?

(Indichi un numero)

Sempre 1
Quasi sempre 2
Molto tempo 3
Una parte del tempo 4
Quasi mai 5
Mai 6

31. Per quanto tempo nelle ultime 4 settimane si è sentita **stanca**?

(Indichi un numero)

Sempre 1
Quasi sempre 2
Molto tempo 3
Una parte del tempo 4
Quasi mai 5
Mai 6

32. Nelle ultime 4 settimane, per quanto tempo la sua salute fisica o il suo stato emotivo hanno interferito nelle sue **attività sociali**, in famiglia, con gli amici?

(Indichi un numero)

Sempre 1
Quasi sempre 2
Una parte del tempo 3
Quasi mai 4
Mai 5

Scelga la risposta che meglio descrive quanto siano **VERE o FALSE** le seguenti affermazioni.

33. Mi pare di ammalarmi un po' più facilmente degli altri

(Indichi un numero)

Certamente vero 1
In gran parte vero 2
Non so 3
In gran parte falso 4
Certamente falso 5

34. La mia salute è come quella degli altri

(Indichi un numero)

Certamente vero 1
In gran parte vero 2
Non so 3
In gran parte falso 4
Certamente falso 5

35. Mi aspetto che la mia salute andrà **peggiorando**

(Indichi un numero)

Certamente vero 1
In gran parte vero 2
Non so 3
In gran parte falso 4
Certamente falso 5

36. Godo di **ottima salute**

(Indichi un numero)

Certamente vero 1
In gran parte vero 2
Non so 3
In gran parte falso 4
Certamente falso 5

37. Nelle ultime 4 settimane, per quanto tempo si è sentita **riposata** al suo risveglio al mattino?

(Indichi un numero)

Sempre 1
Quasi sempre 2
Molto tempo 3
Una parte del tempo 4
Quasi mai 5
Mai 6

38. Nelle ultime 4 settimane, per quanto tempo si è sentita **scoraggiata** a causa della sua salute?

(Indichi un numero)

Sempre 1
Quasi sempre 2
Molto tempo 3
Una parte del tempo 4
Quasi mai 5
Mai 6

39. Nelle ultime 4 settimane, per quanto tempo si è sentita **frustrata** a causa della sua salute?

(Indichi un numero)

Sempre 1
Quasi sempre 2
Molto tempo 3
Una parte del tempo 4
Quasi mai 5
Mai 6

40. Nelle ultime 4 settimane, per quanto tempo si è sentita **preoccupata** a causa della sua salute?

(Indichi un numero)

Sempre 1
Quasi sempre 2
Molto tempo 3
Una parte del tempo 4
Quasi mai 5
Mai 6

41. Nelle ultime 4 settimane, per quanto tempo si è sentita **oppressa** a causa della sua salute?

(Indichi un numero)

Sempre 1
Quasi sempre 2
Molto tempo 3
Una parte del tempo 4
Quasi mai 5
Mai 6

42. Nelle ultime 4 settimane, per quanto tempo ha provato **difficoltà di concentrazione e di ragionamento**?

(Indichi un numero)

Sempre 1
Quasi sempre 2
Molto tempo 3
Una parte del tempo 4
Quasi mai 5
Mai 6

43. Nelle ultime 4 settimane, per quanto tempo ha trovato difficile mantenere la sua **attenzione** a lungo durante lo svolgimento di una attività?

(Indichi un numero)

Sempre 1
Quasi sempre 2
Molto tempo 3
Una parte del tempo 4
Quasi mai 5
Mai 6

44. Nelle ultime 4 settimane, per quanto tempo ha avuto difficoltà a **ricordare**?

(Indichi un numero)

Sempre 1
Quasi sempre 2
Molto tempo 3
Una parte del tempo 4
Quasi mai 5
Mai 6

45. Nelle ultime 4 settimane, per quanto tempo altre persone (familiari o amici) le hanno fatto notare che ha difficoltà a **ricordare ed a concentrarsi**?

(Indichi un numero)

Sempre 1
Quasi sempre 2
Molto tempo 3
Una parte del tempo 4
Quasi mai 5
Mai 6

Le prossime domande riguardano la sua attività sessuale ed il suo grado di soddisfazione. Risponda a ciascuna domanda scegliendo la risposta che più si avvicina al suo caso. Consideri solo le ultime 4 settimane.

46. Nelle ultime 4 settimane, in che misura la **mancanza di stimoli sessuali** ha rappresentato un problema per lei?

(Indichi un numero)

Nessun problema 1
In piccola parte un problema 2
In parte un problema 3
In gran parte un problema 4

47. Nelle ultime 4 settimane, in che misura **l'insufficiente lubrificazione** ha rappresentato un problema per lei?

(Indichi un numero)

Nessun problema 1
In piccola parte un problema 2
In parte un problema 3
In gran parte un problema 4

48. Nelle ultime 4 settimane, in che misura la difficoltà nel **raggiungere l'orgasmo** ha rappresentato un problema per lei?

(Indichi un numero)

Nessun problema 1
In piccola parte un problema 2
In parte un problema 3
In gran parte un problema 4

49. Nelle ultime 4 settimane, in che misura la capacità di **soddisfare sessualmente il partner** ha rappresentato un problema per lei?

(Indichi un numero)

Nessun problema 1
In piccola parte un problema 2
In parte un problema 3
In gran parte un problema 4

50. In generale, quale è stato il suo livello di **soddisfazione rispetto alla sua attività sessuale** nelle ultime 4 settimane?

(Indichi un numero)

Molto soddisfatta 1
Abbastanza soddisfatta 2
Né soddisfatta né insoddisfatta 3
Piuttosto insoddisfatta 4
Molto insoddisfatta 5

51. Nelle ultime 4 settimane, i **disturbi urinari o intestinali** le hanno impedito di svolgere le sue normali attività di relazione con i familiari, con gli amici, con i vicini o nei gruppi di cui fa parte?

(Indichi un numero)

Per nulla 1
Leggermente 2
Un po' 3
Molto 4
Moltissimo 5

52. Nelle ultime 4 settimane, in che misura la sua vita è stata compromessa dal **dolore fisico**?

(Indichi un numero)

Per nulla 1
Leggermente 2
Un po' 3
Molto 4
Moltissimo 5

53. In termini generali, come giudicherebbe la qualità della sua vita?

(Indichi un numero nella scala)

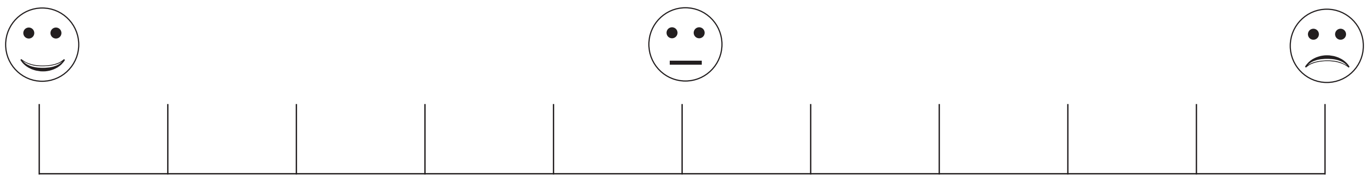

10 9 8 7 6 5 4 3 2 1 0

Migliore qualità della vita

Peggiore qualità della vita possibile (simile alla morte o peggio)

54. Quale dei seguenti termini descrive meglio come si sente se pensa alla sua vita, nel suo insieme?

(Indichi un numero)

Malissimo 1
Scontenta 2
In gran parte insoddisfatta 3
Tanto soddisfatta quanto insoddisfatta allo stesso modo 4
In gran parte soddisfatta 5
Contenta 6
Benissimo 7

MSQOL-54

1998/M

Edizione Italiana

IQOLA SF-36 Italian Version 1.6: Copyright© New England Medical Center Hospitals Inc., 1992. All rights reserved.

Modulo specifico MSQOL-54, versione originale B. Vickrey, 1995; versione Italiana A. Solari, Istituto Nazionale Neurologico C. Besta, Via Celoria 11, 20133 Milano
1998 (1)

ISTRUZIONI: Il questionario intende valutare cosa lei pensa della sua salute. Le informazioni raccolte permetteranno di essere aggiornati su come si sente e su come riesce a svolgere le sue attività' consuete.

Risponda a ciascuna domanda del questionario indicando la sua risposta come mostrato di volta in volta. Se non si sente certo della risposta, effettui la scelta che comunque le sembra migliore.

1. In generale, direbbe che la sua salute è:

(Indichi un numero)

Eccellente 1
Molto buona 2
Buona 3
Passabile 4
Scadente 5

2. <u>Rispetto ad un anno fa</u>, come giudicherebbe, <u>ora</u>, la sua salute in generale?

(Indichi un numero)

Decisamente migliore adesso rispetto ad un anno fa 1
Un po' migliore adesso rispetto ad un anno fa 2
Più o meno uguale rispetto ad un anno fa 3
Un po' peggiore adesso rispetto ad un anno fa 4
Decisamente peggiore adesso rispetto ad un anno fa 5

Le seguenti domande riguardano alcune attività che potrebbe svolgere nel corso di una qualsiasi giornata. La sua salute la limita <u>attualmente</u> nello svolgimento di queste attività?

3. La sua salute la limita attualmente nello svolgimento di **attività fisicamente impegnative**, come correre, sollevare oggetti pesanti, praticare sport faticosi?

(Indichi un numero)

SI, mi limita parecchio 1
SI, mi limita parzialmente 2
NO, non mi limita per nulla 3

4. La sua salute la limita attualmente nello svolgimento di **attività di moderato impegno fisico**, come spostare un tavolo, usare l'aspirapolvere, giocare a bocce o fare un giretto in bicicletta ?

(Indichi un numero)

SI, mi limita parecchio 1
SI, mi limita parzialmente 2
NO, non mi limita per nulla 3

5. La sua salute la limita attualmente nel **sollevare o portare le borse della spesa**?

(Indichi un numero)

SI, mi limita parecchio 1
SI, mi limita parzialmente 2
NO, non mi limita per nulla 3

6. La sua salute la limita attualmente nel **salire qualche piano di scale**?

(Indichi un numero)

SI, mi limita parecchio 1
SI, mi limita parzialmente 2
NO, non mi limita per nulla 3

7. La sua salute la limita attualmente nel **salire un piano di scale**?

(Indichi un numero)

SI, mi limita parecchio 1
SI, mi limita parzialmente 2
NO, non mi limita per nulla 3

8. La sua salute la limita attualmente nel **piegarsi, inginocchiarsi o chinarsi**?

(Indichi un numero)

SI, mi limita parecchio 1
SI, mi limita parzialmente 2
NO, non mi limita per nulla 3

9. La sua salute la limita attualmente nel **camminare per un chilometro**?

(Indichi un numero)

SI, mi limita parecchio 1
SI, mi limita parzialmente 2
NO, non mi limita per nulla 3

10. La sua salute la limita attualmente nel **camminare per qualche centinaia di metri**?

(Indichi un numero)

SI, mi limita parecchio 1
SI, mi limita parzialmente 2
NO, non mi limita per nulla 3

11. La sua salute la limita attualmente nel **camminare per circa cento metri**?

(Indichi un numero)

SI, mi limita parecchio 1
SI, mi limita parzialmente 2
NO, non mi limita per nulla 3

12. La sua salute la limita attualmente nel **fare il bagno o vestirsi da solo**?

(Indichi un numero)

SI, mi limita parecchio 1

SI, mi limita parzialmente 2

NO, non mi limita per nulla 3

Nelle <u>ultime 4 settimane</u>, ha riscontrato i seguenti problemi sul lavoro o nelle altre attività quotidiane, a causa della sua salute fisica?
Risponda SI o NO a ciascuna domanda.

13. Nelle ultime 4 settimane, a causa della sua salute fisica ha ridotto **il tempo dedicato al lavoro o ad altre attività**?

(Indichi per ogni domanda il numero 1 o 2)

SI 1

NO 2

14. Nelle ultime 4 settimane, a causa della sua salute fisica ha reso **meno di quanto avrebbe voluto**?

(Indichi per ogni domanda il numero 1 o 2)

SI 1

NO 2

15. Nelle ultime 4 settimane, a causa della sua salute fisica ha dovuto **limitare alcuni tipi di lavoro o altre attività**?

(Indichi per ogni domanda il numero 1 o 2)

SI 1

NO 2

16. Nelle ultime 4 settimane, a causa della sua salute fisica ha avuto **difficoltà nell'eseguire il lavoro o altre attività** (ad esempio, ha fatto più fatica?)

(Indichi per ogni domanda il numero 1 o 2)

SI 1

NO 2

Nelle ultime 4 settimane, ha riscontrato i seguenti problemi sul lavoro o nelle altre attività quotidiane, a causa del suo stato emotivo (quale il sentirsi depresso o ansioso)?
Risponda SI o NO a ciascuna domanda.

17. Nelle ultime 4 settimane, a causa del suo stato emotivo ha ridotto il **tempo dedicato al lavoro o ad altre attività**?

(Indichi per ogni domanda il numero 1 o 2)

SI 1

NO 2

18. Nelle ultime 4 settimane, a causa del suo stato emotivo ha **reso meno di quanto avrebbe voluto**?

(Indichi per ogni domanda il numero 1 o 2)

SI 1

NO 2

19. Nelle ultime 4 settimane, a causa del suo stato emotivo ha avuto **un calo di concentrazione** sul lavoro o in altre attività?

(Indichi per ogni domanda il numero 1 o 2)

SI 1

NO 2

20. Nelle ultime 4 settimane, in che misura la sua salute fisica o il suo stato emotivo hanno **interferito con le normali attività sociali** con la famiglia, gli amici, i vicini di casa, i gruppi di cui fa parte?

(Indichi un numero)

Per nulla 1

Leggermente 2

Un po' 3

Molto 4

Moltissimo 5

21. Quanto **dolore fisico** ha provato nelle ultime 4 settimane?

(Indichi un numero)

Nessuno 1

Molto lieve 2

Lieve 3

Moderato 4

Forte 5

Molto forte 6

22. Nelle ultime 4 settimane, in che misura il **dolore l'ha ostacolata** nel lavoro che svolge abitualmente (sia in casa sia fuori casa)?

(Indichi un numero)

Per nulla 1

Molto poco 2

Un po' 3

Molto 4

Moltissimo 5

Le seguenti domande si riferiscono a come si è sentito nelle ultime 4 settimane.
Risponda a ciascuna domanda scegliendo la risposta che più si avvicina al suo caso.

23. Per quanto tempo nelle ultime 4 settimane si è sentito **vivace e brillante**?

(Indichi un numero)

Sempre 1
Quasi sempre 2
Molto tempo 3
Una parte del tempo 4
Quasi mai 5
Mai 6

24. Per quanto tempo nelle ultime 4 settimane si è sentito **molto agitato**?

(Indichi un numero)

Sempre 1
Quasi sempre 2
Molto tempo 3
Una parte del tempo 4
Quasi mai 5
Mai 6

25. Per quanto tempo nelle ultime 4 settimane si è sentito così **giù di morale** che niente avrebbe potuto tirarla su?

(Indichi un numero)

Sempre 1
Quasi sempre 2
Molto tempo 3
Una parte del tempo 4
Quasi mai 5
Mai 6

26. Per quanto tempo nelle ultime 4 settimane si è sentito **calmo e sereno**?

(Indichi un numero)

Sempre 1
Quasi sempre 2
Molto tempo 3
Una parte del tempo 4
Quasi mai 5
Mai 6

27. Per quanto tempo nelle ultime 4 settimane si è sentito **pieno di energia**?

(Indichi un numero)

Sempre 1
Quasi sempre 2
Molto tempo 3
Una parte del tempo 4
Quasi mai 5
Mai 6

28. Per quanto tempo nelle ultime 4 settimane si è sentito **scoraggiato e triste**?

(Indichi un numero)

Sempre 1
Quasi sempre 2
Molto tempo 3
Una parte del tempo 4
Quasi mai 5
Mai 6

29. Per quanto tempo nelle ultime 4 settimane si è sentito **sfinito**?

(Indichi un numero)

Sempre 1
Quasi sempre 2
Molto tempo 3
Una parte del tempo 4
Quasi mai 5
Mai 6

30. Per quanto tempo nelle ultime 4 settimane si è sentito **felice**?

(Indichi un numero)

Sempre 1
Quasi sempre 2
Molto tempo 3
Una parte del tempo 4
Quasi mai 5
Mai 6

31. Per quanto tempo nelle ultime 4 settimane si è sentito **stanco**?

(Indichi un numero)

Sempre 1
Quasi sempre 2
Molto tempo 3
Una parte del tempo 4
Quasi mai 5
Mai 6

32. Nelle ultime 4 settimane, per quanto tempo la sua salute fisica o il suo stato emotivo hanno interferito nelle sue **attività sociali**, in famiglia, con gli amici?

(Indichi un numero)

Sempre 1
Quasi sempre 2
Una parte del tempo 3
Quasi mai 4
Mai 5

Scelga la risposta che meglio descrive quanto siano **VERE o FALSE** le seguenti affermazioni.

33. Mi pare di ammalarmi un po' più facilmente degli altri

(Indichi un numero)

Certamente vero 1
In gran parte vero 2
Non so 3
In gran parte falso 4
Certamente falso 5

34. La mia salute è come quella degli altri

(Indichi un numero)

Certamente vero 1
In gran parte vero 2
Non so 3
In gran parte falso 4
Certamente falso 5

35. Mi aspetto che la mia salute andrà **peggiorando**

(Indichi un numero)

Certamente vero 1
In gran parte vero 2
Non so 3
In gran parte falso 4
Certamente falso 5

36. Godo di **ottima salute**

(Indichi un numero)

Certamente vero 1
In gran parte vero 2
Non so 3
In gran parte falso 4
Certamente falso 5

37. Nelle ultime 4 settimane, per quanto tempo si è sentito **riposato** al suo risveglio al mattino?

(Indichi un numero)

Sempre 1
Quasi sempre 2
Molto tempo 3
Una parte del tempo 4
Quasi mai 5
Mai 6

38. Nelle ultime 4 settimane, per quanto tempo si è sentito **scoraggiato** a causa della sua salute?

(Indichi un numero)

Sempre 1
Quasi sempre 2
Molto tempo 3
Una parte del tempo 4
Quasi mai 5
Mai 6

39. Nelle ultime 4 settimane, per quanto tempo si è sentito **frustrato** a causa della sua salute?

(Indichi un numero)

Sempre 1
Quasi sempre 2
Molto tempo 3
Una parte del tempo 4
Quasi mai 5
Mai 6

40. Nelle ultime 4 settimane, per quanto tempo si è sentito **preoccupato** a causa della sua salute?

(Indichi un numero)

Sempre 1
Quasi sempre 2
Molto tempo 3
Una parte del tempo 4
Quasi mai 5
Mai 6

41. Nelle ultime 4 settimane, per quanto tempo si è sentito **oppresso** a causa della sua salute?

(Indichi un numero)

Sempre 1
Quasi sempre 2
Molto tempo 3
Una parte del tempo 4
Quasi mai 5
Mai 6

42. Nelle ultime 4 settimane, per quanto tempo ha provato **difficoltà di concentrazione e di ragionamento**?

(Indichi un numero)

Sempre 1
Quasi sempre 2
Molto tempo 3
Una parte del tempo 4
Quasi mai 5
Mai 6

43. Nelle ultime 4 settimane, per quanto tempo ha trovato difficile mantenere la sua **attenzione** a lungo durante lo svolgimento di una attività?

(Indichi un numero)

Sempre 1
Quasi sempre 2
Molto tempo 3
Una parte del tempo 4
Quasi mai 5
Mai 6

44. Nelle ultime 4 settimane, per quanto tempo ha avuto difficoltà a **ricordare**?

(Indichi un numero)

Sempre 1
Quasi sempre 2
Molto tempo 3
Una parte del tempo 4
Quasi mai 5
Mai 6

45. Nelle ultime 4 settimane, per quanto tempo altre persone (familiari o amici) le hanno fatto notare che ha difficoltà a **ricordare ed a concentrarsi**?

(Indichi un numero)

Sempre 1
Quasi sempre 2
Molto tempo 3
Una parte del tempo 4
Quasi mai 5
Mai 6

Le prossime domande riguardano la sua attività sessuale ed il suo grado di soddisfazione. Risponda a ciascuna domanda scegliendo la risposta che più si avvicina al suo caso.
Consideri solo le ultime 4 settimane.

46. Nelle ultime 4 settimane, in che misura la **mancanza di stimoli sessuali** ha rappresentato un problema per lei?

(Indichi un numero)

Nessun problema 1
In piccola parte un problema 2
In parte un problema 3
In gran parte un problema 4

47. Nelle ultime 4 settimane, in che misura la difficoltà nell'avere o nel mantenere **un'erezione** ha rappresentato un problema per lei?

(Indichi un numero)

Nessun problema 1
In piccola parte un problema 2
In parte un problema 3
In gran parte un problema 4

48. Nelle ultime 4 settimane, in che misura la difficoltà nel **raggiungere l'orgasmo** ha rappresentato un problema per lei?

(Indichi un numero)

Nessun problema 1
In piccola parte un problema 2
In parte un problema 3
In gran parte un problema 4

49. Nelle ultime 4 settimane, in che misura la capacità di **soddisfare sessualmente il partner** ha rappresentato un problema per lei?

(Indichi un numero)

Nessun problema 1
In piccola parte un problema 2
In parte un problema 3
In gran parte un problema 4

50. In generale, quale è stato il suo livello di **soddisfazione rispetto alla sua attività sessuale** nelle ultime 4 settimane?

(Indichi un numero)

Molto soddisfatto 1
Abbastanza soddisfatto 2
Né soddisfatto né insoddisfatto 3
Piuttosto insoddisfatto 4
Molto insoddisfatto 5

51. Nelle ultime 4 settimane, i **disturbi urinari o intestinali** le hanno impedito di svolgere le sue normali attività di relazione con i familiari, con gli amici, con i vicini o nei gruppi di cui fa parte?

(Indichi un numero)

Per nulla 1
Leggermente 2
Un po' 3
Molto 4
Moltissimo 5

52. Nelle ultime 4 settimane, in che misura la sua vita è stata compromessa dal **dolore fisico**?

(Indichi un numero)

Per nulla 1
Leggermente 2
Un po' 3
Molto 4
Moltissimo 5

53. In termini generali, come giudicherebbe la qualità della sua vita?

(Indichi un numero nella scala)

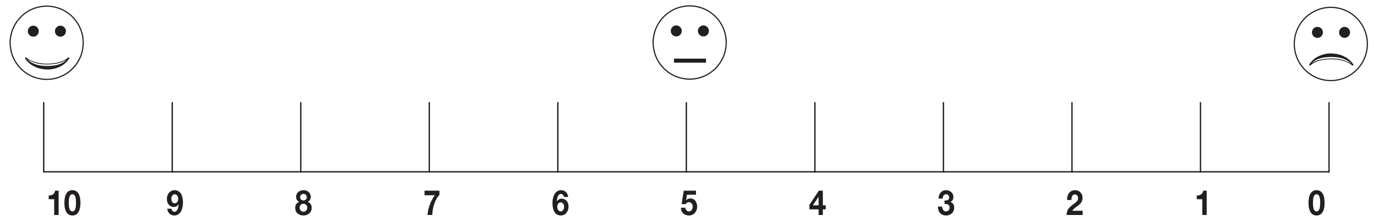

Migliore qualità della vita

Peggiore qualità della vita possibile (simile alla morte o peggio)

54. Quale dei seguenti termini descrive meglio come si sente se pensa alla sua vita, nel suo insieme?

(Indichi un numero)

Malissimo 1
Scontento 2
In gran parte insoddisfatto 3
Tanto soddisfatto quanto insoddisfatto allo stesso modo 4
In gran parte soddisfatto 5
Contento 6
Benissimo 7
